# Coadaptation of Offspring Begging and Parental Provisioning - An Evolutionary Ecological Perspective on Avian Family Life

**DOI:** 10.1371/journal.pone.0070463

**Published:** 2013-07-19

**Authors:** Natalia Estramil, Marcel Eens, Wendt Müller

**Affiliations:** Department of Biology-Ethology, University of Antwerp, Antwerp, Belgium; University of Jyväskylä, Finland

## Abstract

Offspring begging and parental provisioning are the two central social behaviours expressed during the period of parental care. Both behaviours influence each other and it is, therefore, hypothesized that they should ultimately become (genetically) correlated, stabilized by fitness costs to parents and/or offspring. By reciprocally exchanging entire clutches in canaries (*Serinus canaria*), we tested (1) whether there is covariation between these behaviours and (2) whether a mismatch - as introduced by cross-fostering - entails costs. Begging was scored in a standardized begging test and parental provisioning was measured via (a) the actual feeding rate and (b) using the growth rate of the foster nestlings as a proxy. Costs were established in terms of future reproductive investment in subsequent clutches and offspring growth. We found a positive and significant phenotypic covariation between offspring begging and parental feeding when using the growth rate as a proxy and, to a lesser extent, in case of the parental feeding rate. Female parents suffered no future reproductive costs when feeding foster nestlings that were more demanding than their own nestlings. Neither growth measured amongst all offspring nor the reproductive investment measured amongst the female offspring as adults was influenced by their begging behaviour. However, the reproductive investment of female offspring tended to depend on the parental qualities of their foster parents. Thus, offspring may only be able to extract resources within the limit of generosity of their foster parents. This suggests parental control of feeding, which is also supported by the positive covariation between offspring begging and parental feeding.

## Introduction

The family, which is defined as a social unit consisting of one or two parents and their offspring, forms a social environment with significant consequences for individual fitness and trait evolution. Social interactions between parents and offspring as expressed in the family context are of particular interest, since these traits are not only target of selection but can also act as agent of selection by exerting a selective pressure on the trait expression of other family members [1]–[3]. Consequently both the social trait expressed and the social environment can evolve [2]. Studying the expression of behaviours during parent-offspring interactions between parents and offspring and their (co-)evolution has consequently become an important line of research (e.g. [4]–[6]).

Within the family environment, offspring solicitation displays and parental provisioning are probably the most commonly studied behaviours, particularly in birds, insects and mammals [7]. In birds, for instance, parents provide food in response to the begging behaviour of their offspring while offspring adjust their begging behaviour according to their need, quality or the amount of food they receive [8], [9]. Offspring begging and parental feeding have a heritable basis [10]–[14] and are thus able to respond to selection. However, their evolution may not be independent, as the expression of each of these traits depends on the expression of the other. That is, parental provisioning is influenced by offspring begging and parental provisioning alters the begging behaviour of the offspring. As both behaviours affect each other and exert a selective pressure on the expression of the other, quantitative genetic models predict that offspring begging and parental provisioning should become coadapted and ultimately genetically correlated [1], [15]. The genetic correlations are expected to be mostly positive when parents control provisioning and selection predominantly acts on offspring begging (as has been found in birds [6], [10], [16], mice [4], [17] and burying beetles [2], [18] and mostly negative when offspring control parental provisioning and selection predominantly acts on parental provisioning (as has been found in burrower bugs [19]). There is some evidence that these correlations are truly genetic [4], [17]. However, a phenotypic covariation of parental provisioning and offspring begging may also depend on (prenatal) maternal effects [10], [16], [20].

The degree and architecture of covariance is further complicated by an evolutionary conflict of interest between the parents and their young, since parents and offspring rate the costs and benefits of parental investment differently [21]. Typically offspring, which depend on the energetic resources provided by the parents, demand more resources from their parents than their parents are selected to give [21], [22]. It is, therefore, of interest to establish whether the degree of coadaptation represents the optimum for parents or offspring [7], which depends on whether offspring or parents exert behavioural control on the level of food provisioning [6], [15]. Ultimately, such coadaptation should be stabilized by fitness costs either to parents and/or offspring (e.g. [1], [15]). Conversely, the evolutionary resolution of parent-offspring conflict may also depend on the genetic covariance between offspring begging and parental provisioning, for instance in a runaway process [10], [23], [24].

In birds, the available evidence for coadaptation, which is mainly based on cross-fostering studies, is still limited. No evidence for a phenotypic covariation has been reported for the house sparrow (*Passer domesticus*) [12]. A positive covariation has been found in great tits (*Parus major*) and canaries (*Serinus canaria*) [6], [10], [16] suggesting that parents, at least in these two species, control feeding, while selection predominantly acts on offspring begging. Interestingly, the phenotypic covariation between the feeding response of great tit parents and the begging intensity of their biological nestlings was only expressed in the maternal line [10]. In the canary study, the nestling growth rates were used as a proxy for parental feeding rate [16] and it is still unknown whether there is covariation on the behavioural level and, more importantly, whether it differs between male and female parents. Furthermore, little attention has as yet been paid to the question how this coadaptation is stabilized by fitness costs to parents and/or offspring [1], [15]. An exception to this is a recent study showing that female canaries laid fewer eggs when they had raised foster nestlings that begged more intensively than their biological offspring and vice versa. In addition, foster nestlings grew less well when their begging behaviour differed from that of the biological nestlings [6]. Even though both parties apparently paid a cost for a mismatch between begging and parental feeding, these costs appeared to be higher for the offspring as revealed in a second experiment [6]. However, it is not yet known whether and how a mismatch between these behaviours impinges on the future reproductive investment of the offspring.

Using domestic canaries (*Serinus canaria*) as a model species we applied a reciprocal cross-fostering in order to establish a phenotypic covariation between offspring begging and parental feeding, and to test for differences in this relationship between males and females. Canaries are highly suitable to test predictions made by parent-offspring theory as they breed easily in captivity. Both males and females provide food to the offspring, parents increase their feeding rate in response to increasing begging demands [25] and begging is costly [26]. Furthermore, we studied potential costs to parents and offspring that stabilize such coadaptation with a focus on the females, because the costs to mothers and daughters, in terms of fecundity (clutch size/mass) can both be measured in the same way, which allows for a direct comparison.

## Materials and Methods

### Ethics statement

The experiments reported here comply with the current Flemish and Belgian institutional laws and were performed under licenses of Ethical Committee on animal experimentation (ECD) of the University of Antwerp [experiment specific licenses 2008-26 (2010), 2011-07 (2011)]. No deterioration of condition or abnormalities in appearance or behaviour as consequence of the experimental handling (behavioural trials, video recordings, bird measurements and handling) were observed. During reproduction, birds were kept as detailed in this section. In between, birds of the parental generation were kept in large single-sex outdoor aviaries, while their offspring was kept in single-sex indoor aviaries at room temperature (19–24°C) and under a natural light: dark regime.

### Birds and housing condition

In February 2010, we moved approximately two hundred canaries from our own outbred breeding population to single-sex indoor aviaries (2*2*2 m^3^). They were kept on a long day photoperiod (14∶10 h light∶dark) and room temperature of 19–24°C to stimulate their reproductive activity. Birds had *ad libitum* access to a canary seed mixture (Van Camp, Belgium), cuttlefish bone and fresh water, while egg food (Van Camp, Belgium) was given twice a week. After five to nine weeks, we moved the birds to their breeding cages (50*64*40 cm^3^, GEHU cages The Netherlands) randomly mating each bird with a non-related sexual partner randomly mating each bird with a non-related sexual partner. Due to logistical reasons, we conducted two successive series of experiments: the first group of pairs was established in March (N = 37 pairs) and the second in April (N = 59 pairs). During the experiments, each pair occupied a single breeding cage, which was supplied with a nest box, nesting material, food (see above) and water. After the nestlings had hatched, germinated seeds and egg food were given on a daily basis. Parents and nestlings were moved to single-sex indoor aviaries (2*2*2 m^3^) when the nestlings were independent (day 30 after hatching).

### Experimental design

Nests were checked for eggs daily from the day the pairs were formed onwards. Eggs were marked with a non-toxic pen for recognition and replaced with dummy eggs. We stored the eggs on a foam tray at room temperature, with the tip or blunt end pointing downwards. The eggs were turned twice a day. We synchronized hatching within broods to minimize within-brood differences in begging, growth and survival. The synchronization may in addition result in a higher competition between - more equal -nestlings (as predicted by the “sibling rivalry hypothesis”: e.g. [27], [28]) and increase the food demand at peak moments (the “peak load reduction hypothesis” e.g. [29], [30]). Both may lead to higher levels of begging and parental provisioning respectively, when compared to asynchronous broods. However, it is unlikely that this changes the interpretation of our results, given also that all nests were treated in the same way, but these considerations are relevant for a comparison with previous or forthcoming studies. Finally, differences between asynchronous and synchronous broods may be most pronounced under harsh conditions (e.g. [31], but see also [32]), while we provide *ad libitum* quantities of food. We returned all eggs on the day the 4^th^ egg (first series of experiments) or 3^rd^ egg (second series of experiments) was laid, as the latter appeared to be sufficient for synchronization. We reciprocally cross-fostered complete clutches between nests to ensure that any observed phenotypic covariation between offspring begging and parental feeding was not attributed to experience with their biological ( =  focal) parents after hatching. Therefore, focal parents raised foster nestlings, while their biological nestlings ( =  focal nestlings) were raised by foster parents. The number of experimental nests was lower than the number of breeding pairs due to reproductive failures, small clutch size or the lack of a matching partner nest. At hatching (day  = 0) nestlings were weighed and coloured with a non-toxic pen for individual recognition. The modal brood size was 4 (range 2–4). Nestling weight was measured daily until day 13.

Here we present data on 34 nests (N = 17 dyads) (first series of experiments: N = 12, second series of experiments: N = 22).

### Offspring begging intensity

Begging tests were performed on day five after hatching, when the nestlings still do not show a fear response, following a previously established protocol [16], [33] with slight modifications. Briefly, before testing, each focal nest was removed from its cage and all nestlings were fed until satiation with Orlux Handmix (Orlux Versele, Laga Belgium). We selected two intermediate nestlings per nest, and placed each of them individually in a wooden test box (10*10*13 cm^3^) and returned the nest with the remaining nestlings to their original cage. We completely removed the food from the cage 15 min before starting each trial to ensure that the remaining nestlings were not fed while focal nestlings were tested. Each wooden box had two lids, enabling us to open the top and the upper half of the front section of the box when testing. Each box was filled with expanded polystyrene in the lower half. This filling had a central hole (2 cm diameter, 1.5 cm deep) where the nestling was placed. Once a nestling was placed in the central hole, the lids were closed. Each box was moved to a heated climate room (29–30°C) and placed on a separate table (since the vibrations of tapping and the light and vibration stimulus when opening a given box - rather than the (very soft) begging calls of other tests elicit begging, NE pers. observation). After 60 min of food deprivation, the box was opened and we immediately tapped the box three times with an iron bar (21 cm, 51.63 g). Each nestling received, therefore, sound and light stimuli once the box was opened. The begging behaviour of all nestlings was video recorded using Sony video cameras (DCR-SX 30). Each trial ended after five seconds without begging. We closed the boxes and repeated the begging test 30 min later (90 min of food deprivation). In most cases, a maximum of eight nestlings were tested at one time. After testing, we returned the nestlings to their original cage, fed all nestlings until satiation with Orlux Handmix and placed the food back in the cage. Videos were analysed using Windows media player. Nestling begging intensity after 60 and 90 min of food deprivation was estimated by giving a score every second (0 =  not begging; 1 =  gape open; 2 =  gape open, head back; 3 =  gape open, head back and neck stretched; 4 =  as in 3 plus back vertical) and summing up all the scores over the time period, according to [26].

### Parental feeding rate

Parental feeding was measured by using both (i) the actual parental feeding rate during 2 h video observations (ii) the growth rate of the foster nestlings as a proxy for parental feeding behaviour of the focal parents [16]. To this end we tested for a relationship between offspring begging and parental feeding by comparing the begging intensity of the focal nestlings in their foster nest and the parental feeding of focal parents to their foster nestlings sensu [16].

#### Actual parental feeding rate

We recorded the feeding behaviour of the focal parents to their foster nestlings five days after the begging tests, when the nestlings were 10 days old. At this age it is possible to quantify the feeding rates of females and males separately, while the females do the brooding and most of the food transfers at earlier stages. Video recordings were made with Sony cameras (DCR-SX 30), which were installed 30 min before starting each 2 h video session. We removed all food from the cages as soon as the cameras were set. Fresh egg food and germinated seeds were provided immediately before starting the video recording in order to stimulate parents to feed. Nests were video recorded between 10:00 am and 16:00 pm. We weighed the nestlings before and after each trial. Videos were analysed with The Observer XT 10 (Noldus, The Netherlands).

It is difficult to estimate the exact amount of delivered food in granivorous species since parent can vary the amount of food with each food transfer [34]. But the number of feeds per feeding visit has been shown to be a good estimate of parental effort in granivorous bird species in captivity (e.g. [34]). Similarly, the total number of dips has been shown to correlate with mass gain in canaries, indicating its significance [16] (see also below). We used, therefore, the total number of dips of parents' bills into the nestlings beaks [35] to estimate the parental feeding rate ( =  food transfers) and the respective contribution of each parent for the individual feeding rates. Feeding in canaries consists of direct feeding ( =  when either sex feeds the nestlings directly) and indirect feeding ( =  when food transfer to the nestlings occurs after being fed by the partner, typically the male). The dips that were given to the nestlings by one of their parents after receiving food from their partner were credited as (indirect) dips given by the partner, who did the food preparation and pre-digestion.

#### Growth rate as a proxy of parental feeding behavior

First, we aimed to confirm the use of the growth rate of the foster nestlings as a proxy for parental care in our study population.

To assess whether foster brood growth rates can be used as a proxy of parental feeding rates [16], we used the data obtained in (i). We applied a linear mixed model in R (version 2.10.1, R development core team 2009; www.r-project.org) to test for a relationship between the total number of dips given to 9 and 10 day old nestlings (N = 34 broods) and their body mass gain on that day (from 12am–17pm). The natural logarithm of the number of dips given per hour was the response variable, nestling age and the natural logarithms of body mass gain were included as covariates. Brood identity was used as a random factor. We checked for an interaction between the covariates. Neither the interaction between the covariates (LMM, *F _1,63_* = 0.06, *P* = 0.81) nor nestling age had a significant effect on the number of dips (*F_1,63_* = 1.87, *P* = 0.18).

Body mass gain predicted the number of dips that nestlings received during the 2 h video trails on that day (*F_1,65_* = 10.29, *P* = 0.002), as per [16] (see also [36]). Having confirmed that nestling body mass gain can be used as a proxy of the parental feeding rate, we estimated the mean growth rate per nest from the slopes of simple linear regressions of nestling mass on age for each nestling from day 0 to day 13 and averaged the slopes per nest (all nestlings, 0.936≤*r^2^*≤0.997). Even though nestling growth curve is sigmoidal (e.g. [37]), the slope of a linear regression between days 0 and 13 is a reliable indicator of nestling growth (see also [16]).

### Costs for parents and offspring

Females of the parental generation laid a second clutch immediately after the first clutch in 2010, when the first brood had just fledged. However, we only registered clutch size and clutch mass for the second group of females (April, N = 22). All females laid a clutch immediately after the first was fledged. All surviving females were re-mated with the same partner in 2011 (N = 29) and laid another clutch ( =  third clutch). We checked their nests daily and all eggs were weighed on the day they were laid. The experiments in 2011 were performed under the same conditions as those in 2010. The impact of raising foster nestlings that differed in their begging behaviour from the biological nestlings ( =  focal nestlings) on the future reproductive success of the parents was estimated via the difference in begging intensity between focal and foster nestlings (for all nests) after 90 min of food deprivation. We tested whether these differences in begging impinged on the future reproductive investment (of the female) in terms of total clutch mass and the total number of eggs laid in subsequent clutches.

In case of the nestlings we measured focused on changes in nestling growth and future reproductive investment. Since we were interested in changes to growth as a consequence of the cross-fostering, we first tested whether the growth of the focal nestlings, which were raised by foster parents, was related to the difference in begging between foster nestlings ( =  biological nestlings of the foster parents) and focal nestlings. This approach allows us to study whether focal nestlings that begged more than the foster nestlings also grew at a faster rate and vice versa. Daughters (N = 26) were then randomly mated with non-closely related sexual partners in 2011 to test whether differences in parental feeding between focal and foster parents had an impact on their future reproductive success ( =  measured as total clutch mass and size laid in 2011). To this end, we calculated both i) the difference in the actual feeding rate between foster and focal parents (in 2010) and ii) the difference in growth between foster and focal nestlings (growth rate  =  proxy for parental feeding, in 2010).

### Statistical analyses

Shapiro-Wilk and Bartlett tests were used to analyse the normality and homogeneity of variance, respectively. We tested for a relationship between offspring begging and the actual parental feeding rate of both parents by using a linear mixed model. In this case, mean brood begging intensity of focal nestlings was used as response variable and the total number of dips given by focal and foster parents as covariates. Mean brood begging represents the average value of two nestlings per nest, except in two cases where only a single nestling could be included, and forms a repeated measurement (after 60 and 90 min of food deprivation). Nest identity and dyads were used as random effects with nest identity being nested in dyads. The feeding behaviour of foster parents was included to control for its effect on the begging levels of focal nestlings. Since nestlings were more responsive after 90 min of food deprivation, we applied a similar test by using begging intensity of nestlings at 90 min as the only response variable.

We repeated these analyses (i.e. either by using the begging intensity at 60 and 90 min as repeated measurements or only 90 min) using the total number of dips given by male and females, separately.

A similar linear mixed model was applied to test for a relationship between offspring begging and growth rate ( =  proxy of the parental feeding behaviour) with mean brood begging intensity of focal nestlings (after 60 and 90 min of food deprivation) as response variable and mean growth rate of foster nestlings as covariate. In this case, the mean growth rate of focal nestlings was kept to control for the effect of parental feeding of their foster parents on their begging levels.

Begging at 90 min of food deprivation was used as an estimate for the maximum begging levels for the analyses of parental reproductive costs and offspring growth costs. Differences in begging intensities between focal and foster nestlings indicate changes in demand. Therefore, we applied multiple linear regressions to test whether changes in nestling demands had an effect on the investment of the parents (mothers) in subsequent clutches. Total clutch mass of the second or the third clutch respectively, was used as a dependent variable whereas the difference in begging intensity and the total number of eggs laid in the first clutch were used as covariates, in order to control for intrinsic variation in egg size/egg mass.

Similarly, we applied a generalized linear model (Poisson distribution) by using either the total number of eggs laid in the second or the third clutch as the dependent variable and begging intensity differences and total number of eggs laid in the first clutch as covariates. In two nests, data were only available for clutch size but not for clutch mass.

To test whether focal nestlings pay a cost of begging at a different rate than foster nestlings, we used a simple linear regression with growth rate as the response variable and changes in begging demands as the explanatory variable. We repeated this analysis for the daughters, only. We also applied a simple linear regression to test whether the clutch mass of daughters was affected by changes in begging demands and a generalized linear model (Poisson distribution) to test for this effect on clutch size. No covariation would be expected under stabilizing selection and parental control, if parents do not increase their feeding rate when exposed to more demanding nestlings.

The effect of parental feeding changes on the future investment of the daughters was tested by applying a simple linear regression with total clutch mass laid in 2011 as a response variable. Either the difference in number of dips given by foster and focal parents during the 2 h video recordings or the difference in growth rate between the foster and focal nestlings was used as a covariate. A similar test was applied with clutch size laid in 2011 as response variable (generalized linear model, Poisson distribution).

We use a top-down strategy to obtain the minimal. We use a top-down strategy to obtain the minimal model, starting with the model that contains all fixed explanatory variables and their interactions. When applying nested models, the random structures were compared by using restricted maximum likelihood (REML) estimators while the optimal fixed structure was obtained by using maximum likelihood (ML), as described in [38]. All reported statistical tests were two-tailed and executed in R, version 2.10.1 (R development core team 2009; www.r-project.org). The alpha value was 0.05.

## Results

### Covariation between offspring begging and parental feeding rate

**Figure 1:**
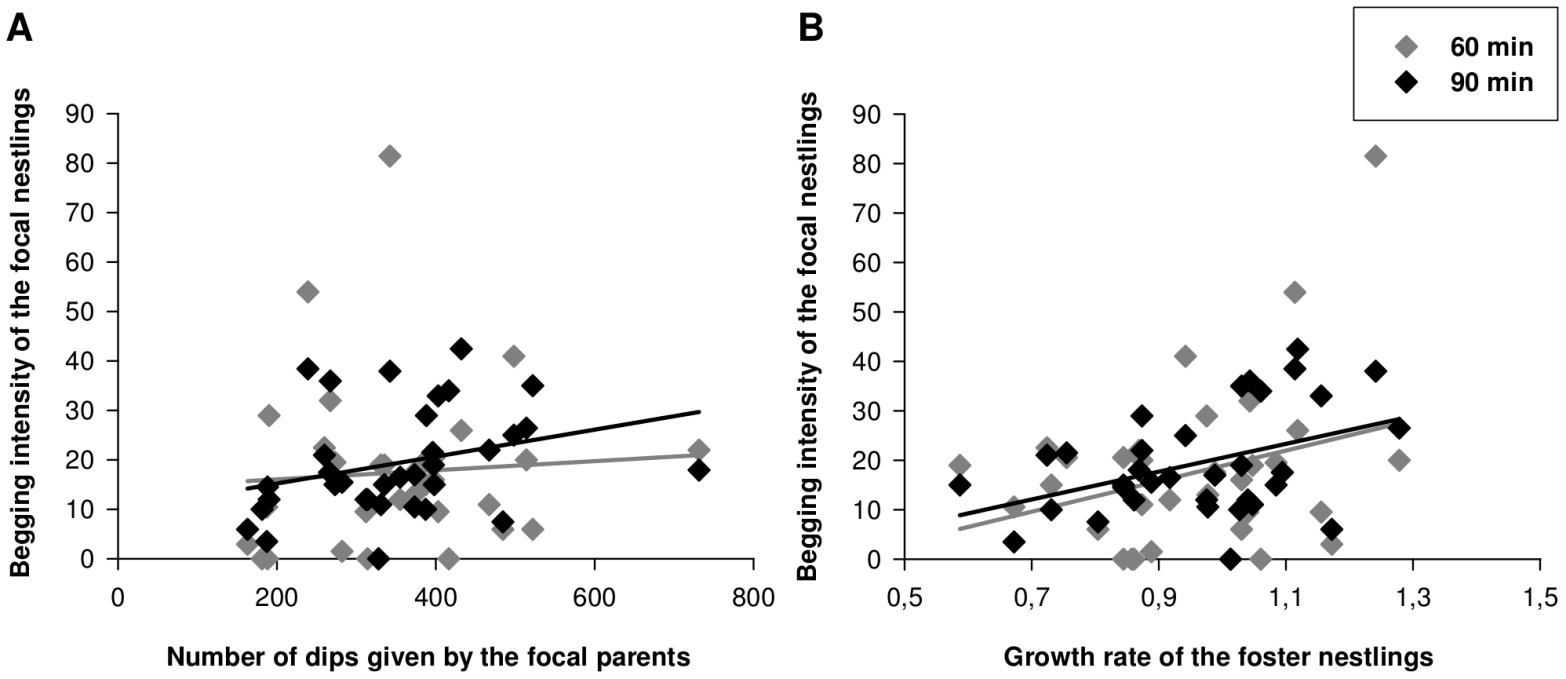
Relationship between begging intensity and parental feeding. The mean brood begging intensity of focal nestlings after 60 (grey) and 90 (black) minutes of food deprivation did not covary significantly with (A) the feeding rate of the focal parents ( =  the total number of dips given by the focal parents to their foster nestlings during a 2 h video session), but with (B) the mean growth rate of the foster nestlings ( = proxy of the intensity of parental feeding of the focal parents).

There was no significant relationship between the begging intensity of focal nestlings and the total number of dips given by the focal ( =  biological) parents to foster nestlings (LMM, *F_1,15_* = 0.64, *P* = 0.44, Figure 1A). Nor was there such a relationship between the begging intensity of focal nestlings and the total number of dips given by their foster parents (*F_1,15_* = 1.03, *P* = 0.33). Removing the term “total number of dips given by foster parents”, which was included to control for the effect of the feeding rates of the foster parents on the begging of the focal nestlings, made little difference to the results (*F_1,16_* = 1.12, *P* = 0.31). However, when using the begging intensity after 90 min of food deprivation (maximum begging level) as the only response variable, we found a positive and significant covariation between the begging intensity of the focal nestlings and the total number of dips given by their biological parents (*F_1,15_* = 4.9, *P* = 0.043).

No significant relationship was found between the begging intensity of focal nestlings and the total number of dips given by the focal father (*F_1,15_* = 0.36, *P* = 0.56) nor with the foster father (*F_1, 15_* = 0.15, *P* = 0.71). Removing the term “total number of dips given by the foster father” made little difference to the results (*F_1,16_* = 0.36, *P* = 0.56). A lack of covariation was also observed between the begging intensity of focal nestlings and the total number of dips given by the focal mother (*F_1,15_* = 0.03, *P* = 0.87) and the foster mother (*F_1,15_* = 2.15, *P* = 0.16). Removing the term “total number of dips given by the foster mother” made little difference to the results (*F_1,16_* = 0.25, *P* = 0.62).

Similarly, we did not find evidence for covariation between the begging intensity of focal nestlings at 90 min of food deprivation and the feeding rate of each parent (focal father: *F_1,15_* = 2.2, *P* = 0.16; focal mother: *F_1,15_* = 0.02, *P* = 0.89). Removing the term “total number of dips given by the foster father” and “total number of dips given by the foster mother”, respectively, made little difference to the results.

### Covariation between offspring begging and the growth rate of the foster nestlings as a proxy for feeding behaviour

The begging intensity of focal nestlings was significantly and positively related to the growth rate of foster nestlings that were raised by the focal parents (LMM, *F_1,15_* = 9.82, *P* = 0.007, Figure 1B). The begging intensity and growth rate of focal nestlings tended to covary negatively, but this was not statistically significant (*F_1,15_* = 3.52, *P* = 0.08). Removing the term “growth rate of focal nestlings”, made little difference to the results (*F_1,16_* = 9.36, *P* = 0.008).

### Costs of the cross-fostering for parents

**Figure 2:**
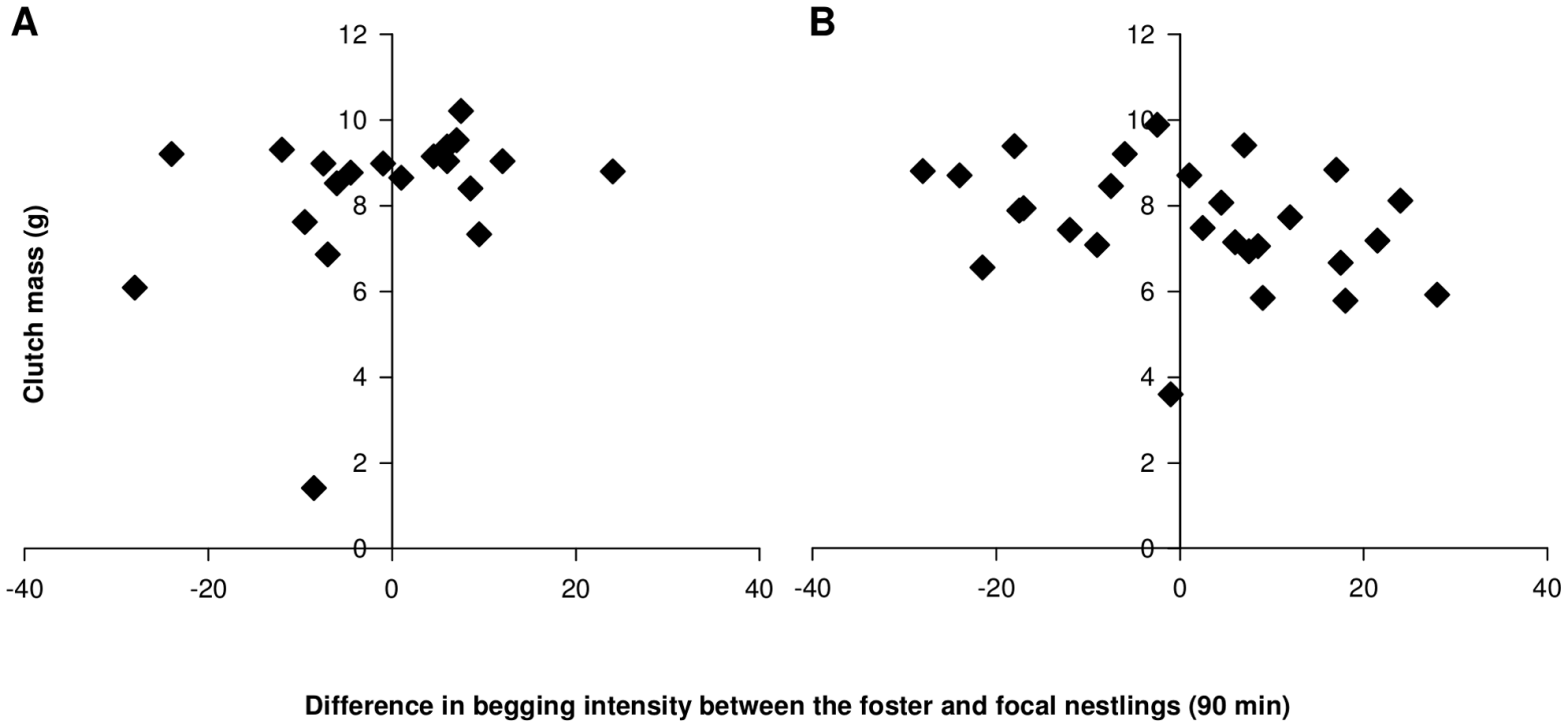
Consequences of the experimentally induced change in begging demands on female investment. The difference in mean brood begging intensities between foster and focal nestlings measured after 90 min of food deprivation did not affect the clutch mass of (A) the second (2010) or (B) the third clutch (2011).

The mass of the second clutch (laid in 2010) was not affected by the experimental change in brood begging intensity, calculated as the difference in begging between the foster nestlings, which were reared by the focal parents and the focal nestlings (LM, *t_16_* = 1.72, *r^2^* = 0.22, *P* = 0.10, Figure 2A). Similarly, the mass of the third clutch (laid in 2011) was not affected by differences in brood begging intensity (LM, *t_24_* = −1.58, *r^2^* = 0.02, *P* = 0.13, Figure 2B). Clutch size was not significantly affected by the changes in begging demands (GLM, second clutch, *z_18_* = −0.07, *P* = 0.94; third clutch, *z_26_* = 0.15, *P* = 0.88).

### Costs of the cross-fostering for offspring

**Figure 3:**
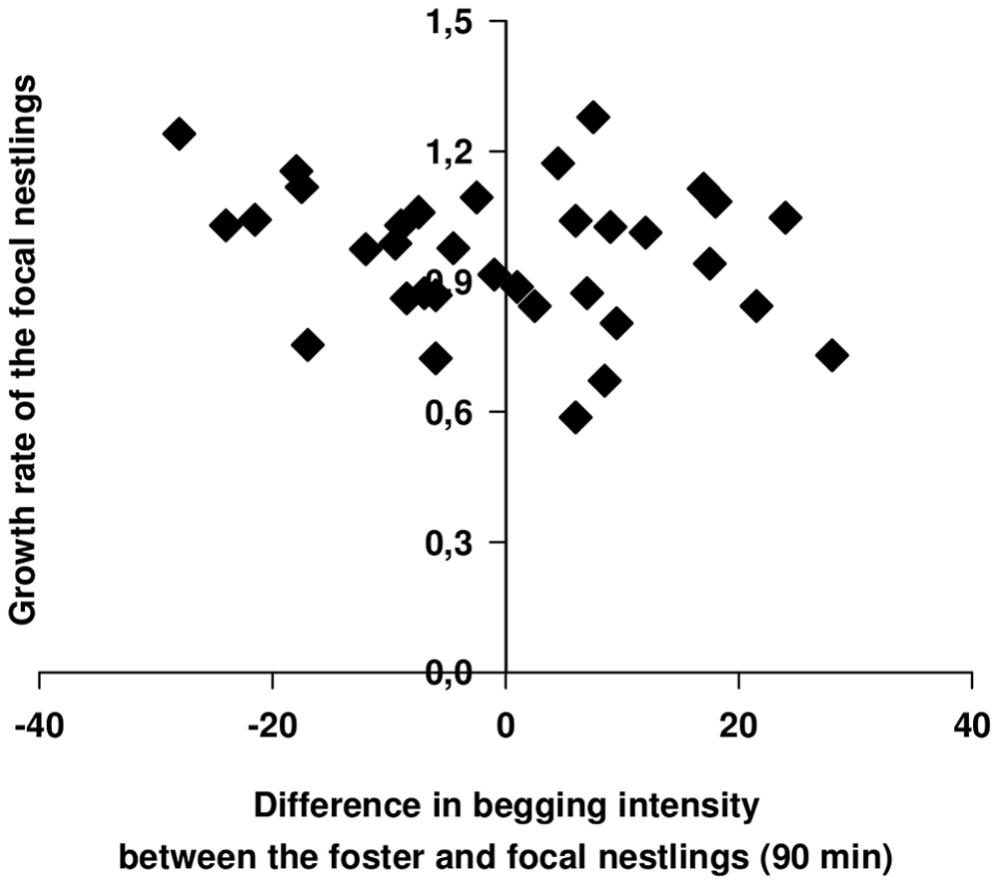
Changes in begging demands and their effects on offspring growth. The difference in mean brood begging intensities between foster and focal broods measured after 90 min of food deprivation had no significant effect on the growth rate of the focal nestlings.

**Figure 4:**
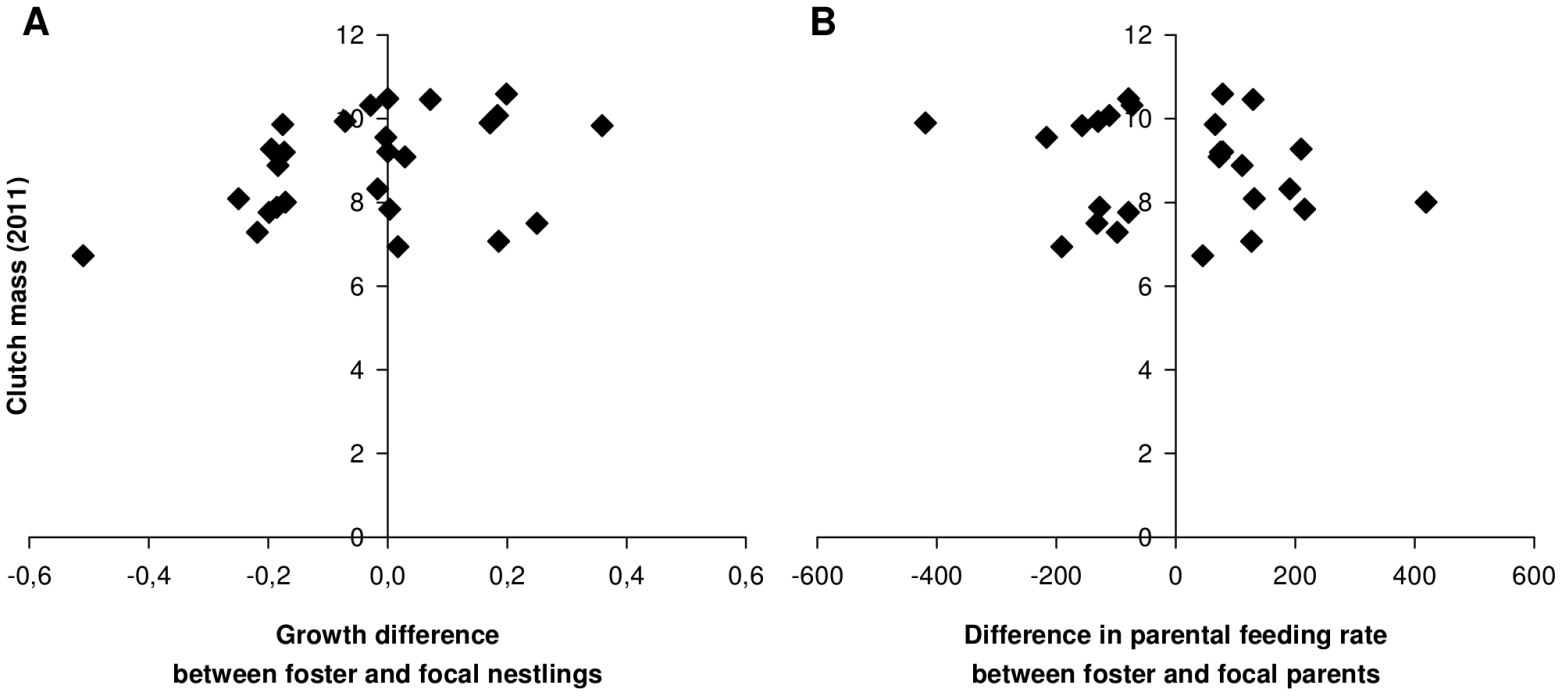
Consequences of a change in parental feeding behaviour on the reproductive investment of the daughters. The total clutch mass (2011) of the daughters tended to be affected by (A) the difference in growth rate between foster and focal nestlings (2010), but not by (B) the difference in feeding rate ( = total number of dips given during a 2 h video session) between foster and focal parents (2010).

There was no effect of the difference in begging between foster and focal nestlings on the growth rate of focal nestlings (LM, *F_1,32_* = 1.76, *r^2^* = 0.02, *P* = 0.19, Figure 3). This was also the case when using only the daughters in the analysis (LM, *F_1,18_* = 0.33, *r^2^* = 0.02, *P* = 0.57). The difference in begging demands did not have significant long-lasting effect on the clutch size (GLM, *z_24_* = 0.01, *P* = 0.99) or clutch mass (LM, *F_1,24_* = 1.04, *r^2^* = 0.042, *P* = 0.32) laid by the daughters in their first breeding season. However, the clutch mass of the daughters tended to be affected by the difference in parental feeding rates between foster and focal parents, when using the difference in growth rate between foster and focal nestlings as a proxy of changes in parental feeding rate (*F_1,24_* = 4.10, *r^2^* = 0.11, *P* = 0.054, Figure 4A). This indicates that daughters tend to lay heavier clutches when they are raised by foster parents feeding at higher rates than focal parents and vice versa. However, no such tendency was observed when using the difference in number of dips between foster and focal parents as explanatory variable (*F_1,24_* = 0.66, *r^2^* = 0.01, *P* = 0.43, Figure 4B). The clutch size of daughters was not significantly affected by the difference in growth rate between foster and focal nestlings ( =  proxy of changes in parental feeding rate) (GLM, *z_24_* = 0.35, *P* = 0.73) or by the difference in number of dips between foster and focal parents (*z_24_* = −0.24, *P* = 0.81).

## Discussion

The family life in species with parental care typically creates a social environment in which traits such as offspring begging and parental feeding that are expressed by one family member exert a selective pressure on the trait expression in another family member. As a result, these traits should ultimately become genetically correlated [1], [15]. Such genetic correlations are expected to be positive when parents control the level of parental provisioning and selection predominantly acts on offspring begging and vice versa in case of a negative correlation. Our experimental evidence is consistent with these theoretical predictions, suggesting that parents control feeding, which is further supported by our results on the stabilizing costs.

### Covariation between offspring begging and parental feeding

We found evidence for a positive and significant phenotypic covariation between offspring begging and parental feeding levels, the latter was estimated via the mean nestling growth rates of the foster nestlings parents reared. Thus, we were able to confirm the existence of a positive covariation between offspring begging and parental feeding, as has recently been reported in canaries [16]. This adds to the evidence and importance of the previous findings, even more so since we used a slightly different experimental design [39], [40]. The positive phenotypic correlation may represent a truly genetic correlation [4], [17], but we cannot rule out that environmental factors before cross-fostering contribute to this relationship. In birds, prime candidates for generating such phenotypic covariation are effects that act via maternally derived egg components. Maternally derived steroid hormones in the yolk of bird eggs have been shown to modulate offspring begging behaviour in a number of bird species including canaries (e.g. [41], reviewed in [42] but see e.g. [35]). Maternal yolk carotenoids transferred into the egg yolks may be another candidate to match offspring demand to parental feeding (e.g. [43], [44]). Our experimental design, however, does not enable us to estimate the relative contributions of genes and other prenatally acting factors any further.

Even though we were able to confirm the outcome of a previous study, indirect measures of parental care were used, as the parental feeding levels were estimated via the growth pattern of the foster nestlings [16]. Thus, it remained to be shown whether these traits correlate at the behavioural level, which will also allow testing whether this relationship differs between males and females. We indeed found a significant relationship between the actual rate of parental feeding (to foster nestlings) and offspring begging, with no evidence for a sex specific pattern (but see [10], [17]). However, the power to detect sex specific relationships on the behavioural level is likely to be lower as the rate of parental feeding is based on fewer feeding visits as it considers only one sex.

However, the observed covariation indicates that the previously established (indirect) relationship also holds at the behavioural level. But this was only the case when considering the begging intensity at the highest level of food deprivation (after 90 min) as response variable. The reasons for this may be multifold:

Offspring growth may in general represent a more robust measure for parental feeding, as it integrates the parental feeding over a period of 13 days. In contrast, parental feeding rates were recorded during 2 h sessions on a particular day, which is obviously a much shorter timeframe. Yet, offspring growth integrates not only a much larger timeframe, but also other aspects such as the parental responsiveness to (changes in) nestlings' food demand, while we control for variation in hunger level during the measurement of parental feeding by standardizing the satiation of the nestlings. Thus, there may be no covariation between offspring begging and actual number of dips at a certain hunger level, but with parental responsiveness to offspring needs [10]. This may be less well captured in our measure of parental feeding during a standardized 2 h observation. Experimental designs in which changes in begging demands are implemented, for instance by changing the brood size or using playbacks of begging nestlings (sensu [10]) could provide useful insights in this respect.

The standardization of both the begging and the parental feeding may also explain why we only find covariation between parental feeding and begging at the highest level of food deprivation. Before the parental feeding behaviour was recorded, nestlings were food deprived, and in contrast to the begging trial, in this case they were not satiated. Furthermore, parents need to pre-digest the food before feeding the nestlings (which can take up to 14 min from the time new food is introduced into the cage, N. Estramil pers. observ.), increasing once more the period of food deprivation. Finally, the begging intensity may have been higher at a given hunger level due to the presence of siblings in the nest. The begging intensity of nestlings during the video-recordings was, therefore, likely comparable to the level of nestlings, which were food deprived for 90 min. Thus, parental feeding may covary with offspring behaviour, when both are measured at similar hunger levels suggesting a close match between offspring demand and the rate of parental feeding.

### Costs to parents and offspring

We investigated potential consequences of a mismatch between parental feeding and offspring begging on the basis of a reciprocal cross-fostering design. Our results suggest that parents control the amount of resources that are provided and that the costs of a mismatch are paid by the offspring:

Firstly, the difference in begging behaviour between foster and focal nestlings did not affect the future reproductive investment of the mothers, neither in terms of total clutch mass or total number of laid eggs within and across years. This is in contrast with a previous study by Hinde and colleagues [6] showing that canary females that raised more demanding nestlings than their own laid fewer eggs in the following year, while the opposite was true for females that raised less demanding foster nestlings. These differences may relate to slightly different experimental designs. The breeding design of Hinde and colleagues [6] was more demanding, with females raising two broods in the first year and laying eggs in the second year of study. Furthermore, the focal nestlings in the previous study were raised by their own parents, while we applied a reciprocal cross-fostering. This may become relevant if the foster parents would influence the begging behaviour of the focal nestlings, which should be taken into account when interpreting the data. The reciprocal design may then lead to smaller differences within dyads. But this remains speculative, and it will require further studies to estimate the potential fitness costs for parents under a number of different conditions. As pointed out above, there is still a lack of studies investigating the costs that may stabilize coadaptation.

Secondly, the growth rate of the focal brood did not vary with the difference in begging intensity between foster and focal brood. Thus, offspring begging at a lower level than the biological nestlings benefited from the higher generosity of their foster parents, while offspring begging more vigorously than the biological nestlings were unable to extract sufficient resources to reach a higher growth rate than low begging nestlings. In fact, they may have been able to obtain slightly more food than the parents initially intended. However, these benefits may vanish due to the costs of begging (e.g. [26], [45], [46]). The lack of long-lasting effects of the difference in begging on the clutch size and clutch mass of the daughters in the subsequent year further supports this argumentation. Alternatively, the difference in begging intensity may be too small to have long-lasting consequences (but see [6]).

Thirdly, daughters suffered from a mismatch between offspring begging and parental feeding in terms of future fecundity. We found a marginally non-significant tendency for a relationship between differences in parental feeding rates ( =  when using the growth rate as a proxy) between foster and focal parents and the future reproductive investment of the daughters. This implies that daughters made a smaller reproductive investment when they were raised by foster parents that were less generous than their biological parents and larger investment when raised by more generous parents. The most likely explanation for this are costs from (unrewarded) high levels of begging, given that there were no direct costs in terms of reduced growth rate (see above). Thus the possibilities to demand additional resources from the parents appear to be limited for the offspring, while the costs of changes in begging demand were smaller for parents than in a previous study [6]. Yet, this is also the first study to measure the costs to mothers and daughters in the same currency (clutch mass/size) within the same experiment, which facilitates the comparison between mothers and daughters. These results show that daughters' investment tend to be more affected by changes in parental feeding than their mothers by changes in begging demands, both suggesting that parents do have the control over feeding rates [6], [15].

In general, our results provide evidence for covariation between offspring begging and parental feeding at the behavioural level and suggest that parents control the feeding rates. These findings may also be taken as evidence for a resolved conflict dictated by selection acting more strongly on offspring begging [15]. However, we did not find direct antagonistic fitness consequences [7]. Conversely, parents may be too powerful for the offspring to manipulate feeding and therefore, this conflict will never emerge at the phenotypic level [7], [47], [48]. More detailed empirical studies on parent-offspring interactions and the fitness costs to both counterparts will be necessary to understand how this covariation is maintained.
