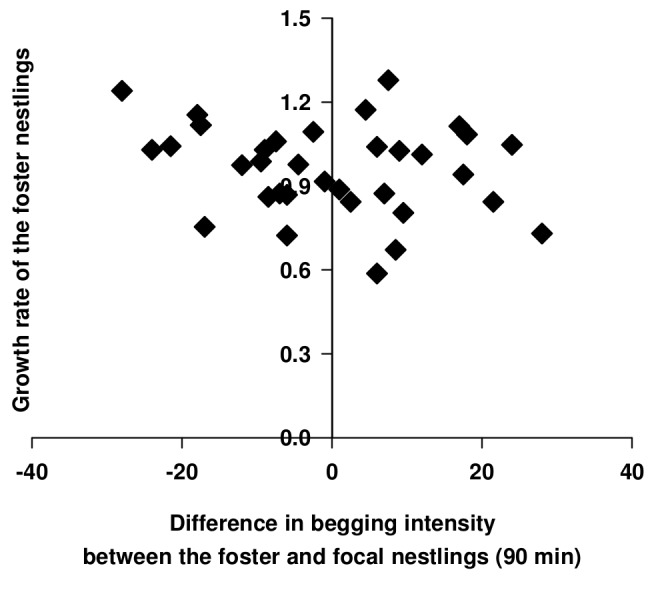# Correction: Coadaptation of Offspring Begging and Parental Provisioning - An Evolutionary Ecological Perspective on Avian Family Life

**DOI:** 10.1371/annotation/cffd172a-2937-4705-aa1f-20de7cb029b0

**Published:** 2013-08-13

**Authors:** Natalia Estramil, Marcel Eens, Wendt Müller

The wrong version of Figure 3 was used in the published article. Please use the following link to download the correct version of this figure:

**Figure:**